# Numerical Analysis of Laminated Veneer Lumber Beams Strengthened with Various Carbon Composites

**DOI:** 10.3390/polym16121697

**Published:** 2024-06-14

**Authors:** Michał Marcin Bakalarz, Paweł Grzegorz Kossakowski

**Affiliations:** Department of Theory of Structures and Building Information Modeling (BIM), Faculty of Civil Engineering and Architecture, Kielce University of Technology, Al. Tysiaclecia Panstwa Polskiego 7, 25-314 Kielce, Poland; mbakalarz@tu.kielce.pl

**Keywords:** Abaqus, deformation, load-bearing capacity, numerical model, stiffness, stress, wood

## Abstract

Among the many benefits of implementing numerical analysis on real objects, economic and environmental considerations are likely the most important ones. Nonetheless, it is also crucial to constrain the duration and space necessary for conducting experimental investigations. Although these benefits are clear, the applicability of such models must be appropriately verified. This research subjected validation of numerical models depicting the behavior of unstrengthened and strengthened laminated veneer lumber (LVL) beams. As a reinforcement, a carbon fiber reinforced polymer (CFRP) sheet and laminates were used. Experiments were conducted on full-scale members within the framework of the so-called four-point bending testing method. Numerical simulations were performed using the Abaqus software. Two types of material models were examined for laminated veneer lumber: linearly elastic and linearly elastic–perfectly plastic with Hill’s yield criterion. A distinction was made in the material properties of carbon composites based on their location on the height of the cross-section. The outlined numerical models accurately depict the behavior of real structural elements. The precision of predicting load-bearing capacity amounts to a few percent for strengthened beams and a maximum of eleven percent for unstrengthened beams. The relative deviation between numerical and experimental values of bending stiffness was at a maximum of seven percent. Applying the elastic–plastic model enables accurate representation of the load versus deflection relation and the distribution of stress and deformation of strengthened beams. Based on the findings, directives were provided for further optimization of the positioning of composite reinforcement along the span of the beam. Reinforcement design of existing laminated veneer lumber members can be made using presented methodology.

## 1. Introduction

The requirement for reinforcing wooden-based structures primarily stems from their degradation and impairment. Impairments and degradation are typically caused by environmental influence (meteorological, biological, chemical, etc.); inherent senescence processes deriving the inception of deficits; human errors made at the phases of design, fabrication, or during the assembly; and improper utilization as well as the absence of relevant inspections and preservation measures [1,2,3]. In addition, it could be associated with other aspects, such as structure optimization or the requirements to prolong their service life. Optimization is related to the minimization of wood utilization ensuing from the employment of smaller cross-sectional dimensions or the inclination towards deploying faster-growth timber species. Prolonging existing structures’ operational lifespan often requires enhancing their load-bearing capacity.

The popular approach to reinforce timber structures involves incorporating an auxiliary component that will synergize with it. Presently, elements fabricated from steel [4,5,6,7,8], aluminum, or composites [9,10,11] are employed for this objective. They are unified via adhesive or mechanical fixtures with the structural element. Considerable focus has been bestowed upon this subject matter. Scientists have described the influence of reinforcement and its placement on the solution’s load-bearing capacity, stiffness, ductility, or economic efficiency. Experimental research is frequently juxtaposed with theoretical [12] or numerical outputs. The following article outlines selected studies from this field, centering primarily on numeric deliberations. The Reader will find the state of the art on reinforcing timber subject matter more extensively in the referenced literature [13,14,15].

Numerical investigations were typically conducted in commercial applications such as Abaqus [16] or Ansys [17]. Various types of material models for timber and composite materials were under examination. It must be emphasized that most of these predominantly focus on combinations of models incorporating elastic or elastic–plastic behavior, either encompassing or excluding the application of Hill’s yield criterion [18]. Hashin’s damage model was implemented in selected scholarly documents [19]. Material models were formulated based on previously conducted experimental research outcomes, manufacturer-provided data, or an amalgamation of both. In simulating wood-to-wood or wood-to-composite connections, options for ideal joints were frequently employed, disregarding the effect of the adhesive joint. An alternative methodology has been delineated in the research conducted by Kawecki and Podgórski [20], among others. They deduced that neglecting the stiffness of adhesive bond line could result in over-projection of the beam’s stiffness. Typically in numerical simulations, loading force and the supporting conditions were determined by rigid bodies that precisely replicate the dimensions of the actual experimental station components. Their engagement with the wooden surface presumes no penetration of bodies and a preliminary assumed friction coefficient.

Kossakowski and Ordysiński [21] confirmed the appropriateness of utilizing elastic models to describe the behavior of wood and composite materials for strengthened timber beams with composite sheets within the elastic range of work. Nowak et al. [22] demonstrated a juxtaposition of experimental and numerical findings for old solid beams strengthened with CFRP laminates. They verified two models of wood behavior—elastic and elastoplastic—whilst considering Hill’s yield criterion. Satisfactory accuracy was achieved between the equilibrium paths within the elastic range of work and the stress distributions within the laminates. Corresponding findings were put forward by Glišović et al. [23] for glued laminated timber beams strengthened with CFRP laminates. The Authors assumed linearly elastic and linearly elastic–perfectly plastic models for the behavior of wood in the tensile and compressive zone, respectively, while considering Hill’s yield criterion. A parametric numerical analysis, considering the impact of the wood species and CFRP composite, was put forward by Kim et al. [24]. In addition to other factors, they analyzed the increments in load-bearing capacity, stiffness, and energy absorption. One of the conclusions drawn is the optimal level of cross-sectional reinforcement ratio, equivalent to 1.9%. Dourado et al. [25] experimentally and numerically evaluated the feasibility of restoring solid timber beams using CFRP composite material. The independent variables under consideration were the thickness of the reinforcement and the technique deployed for adhesive distribution. The “mixed-mode cohesive damage model” was employed in the numerical investigations, achieving considerable precision. The application of synthetic Vectran fibers for strengthening of glue-laminated timber beams was presented in [26]. The Authors described the wood as a “bilinear isotropic hardening” material, postulating a yield point equivalent to 70% of the compressive strength parallel to the grain.

The scholarly literature lacks numerical analysis concerning the reinforcement of full-size elements manufactured from laminated veneer lumber. Hence, this subject matter is elaborated in this study. As a reinforcement, it was determined to adopt CFRP sheets bonded to external surfaces, CFRP laminates bonded in predrilled grooves, and CFRP laminates bonded to the lower surface. Outcomes for two LVL material models—linearly elastic and linearly elastic–perfectly plastic—were presented and juxtaposed with experimental results. A new methodology for simulating composite materials’ characteristics in the compression area has been suggested. This methodology was based on reduction of mechanical properties and the interpolation of the stiffness of the composite constituents. The comprehensive research encompasses the most typical strengthening configurations and basic operational parameters of bent members, such as load-bearing capacity, stiffness, stress distribution, and deformations. The conclusions of the numerical analyses were compared using findings from our own experimental research. This study contributes to the advancement of scientific understanding regarding the functioning of veneer–resin–CFRP composites. Presented numerical models can be used to design reinforcement for existing laminated veneer constructions.

## 2. Materials and Methods

Only the principal assumptions about the conducted experimental investigations were provided here. A detailed description of these can be discovered in the works [27,28,29,30,31,32].

**Figure 1:**
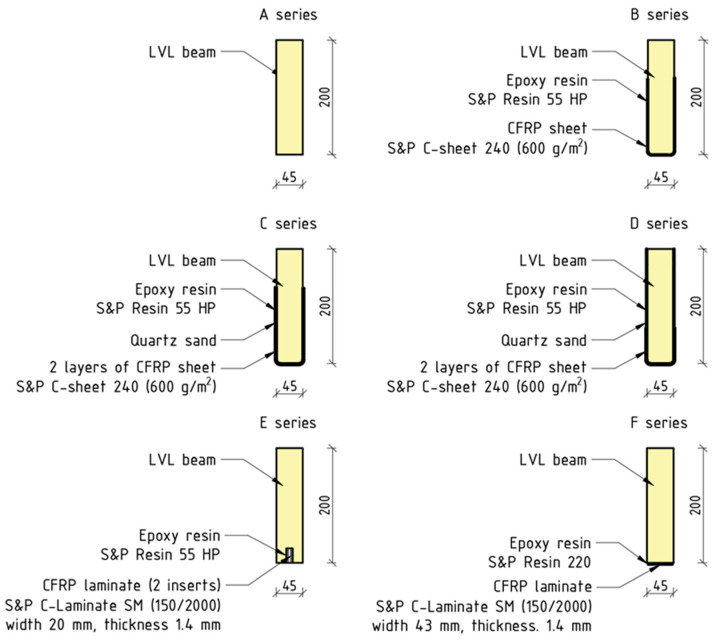
Strengthening configurations [28].

Experimental investigations were conducted on unstrengthened and strengthened laminated veneer lumber beams of nominal dimensions 45 × 200 × 3400 mm by the directives of the PN-EN 408+A1:2012 [33] and PN-EN 14374:2005 [34] standards. The beams were procured from the entity Steico Sp. z o.o. (Czarnków, Poland) [35]. Unstrengthened beams were denoted as A-series. The S&P C-Sheet 240 unidirectionally reinforced carbon sheets [36] and S&P C-Laminate carbon laminates [37] were used as the reinforcement. With a breadth of 300 mm, the sheets were bonded to the exterior surfaces following a U-configuration schema (research series B, C, and D) as shown in Figure 1. Carbon laminates with dimensions of 1.4 × 20 mm were bonded into grooves (E-series) with dimensions of 11 × 25 mm, achieving so-called near-surface mounted (NSM) reinforcement. Carbon laminates of 1.4 × 43 mm were bonded to the bottom surface (F-Series) as shown in Figure 1. Epoxy-based adhesives S&P Resin 55 HP [38] and S&P Resin 220 [39] were used for the adhesion of reinforcements to structural members. 

**Figure 2:**
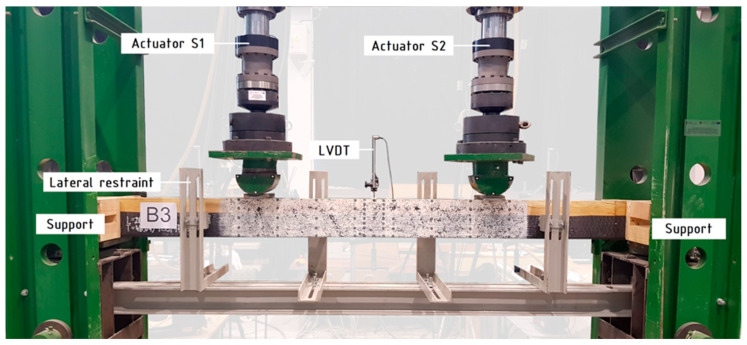
View of test stand [28].

The view of the test stand is shown in Figure 2. The static scheme of the so-called four-point bending test was implemented. The distance between concentrated forces was equivalent to six times the height of the cross-section, 1200 mm. The distance from the concentrated force axis to the nearest support axis was equal to 4.5 times the height of cross-section, 900 mm. A steel guide plate of 10 mm in thickness and 100 mm in width, situated on the supports and at the place of applied concentrated forces, was employed to spread the load across a wider surface. Additional lateral restraints were used to prevent the movement of the beam perpendicularly to bending plane. The deflection was measured at the midspan at the upper surface via an inductive linear variable displacement transducer (LVDT). The applied force was regulated using the displacement speed of the hydraulic actuators; a 7 mm/min loading rate was selected. The bending process was executed until the beam suffered irreversible damage. The failure of the beam was associated with damaging of the laminate veneer lumber or composite material, followed by a substantial decrease in bending strength.

## 3. Numerical Analysis

Numerical analysis was conducted using the Standard module of Abaqus 2017 software [16]. An individual numerical model was prepared for each research series. Owing to the bisymmetry of the system, only a quarter of the beam was modelled to decrease the computational time. The translational degrees of freedom U1, U2, and U3 correspond to the global coordinate system’s x, y, and z axes. The rotations R1, R2, and R3 are analogously correlated. The principal component of the numerical analyses involved the validation of two material models for laminated veneer lumber: (1) linearly elastic and (2) linearly elastic–perfectly plastic, taking into account Hill’s yield criterion.

### 3.1. Model Description

**Figure 3:**
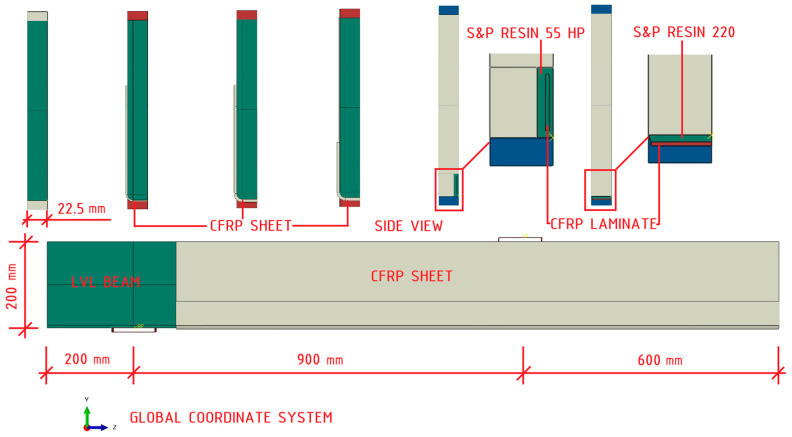
Assembled model details.

Eight constituent parts were created, including: laminated veneer lumber (LVL) beam, adhesive utilized within the groove, adhesive applied on the bottom surface, two sections representing the carbon fiber reinforced polymer (CFRP) sheet, CFRP laminate bonded within the groove, CFRP laminate bonded to the exterior surface, and the steel guide plate. The wooden beam was modelled as a three-dimensional deformable body by extruding a rectangular sketch with dimensions of 22.5 × 200 mm over 1700 mm. Specifics encompassing the groove (5.75 × 25 mm) and the rounding of corners (radius 6.25 mm) were made by extruding the cuts to a distance of 1420 mm. These values correspond to the inventoried values of the processed segments of the beams. The adhesives were represented as deformable three-dimensional bodies with dimensions of 5.75 × 25 × 1420 mm (groove) and 22.5 × 2.5 × 1420 mm (external application). The dimensions of the CFRP laminates were 1.4 × 10 × 1400 mm and 21.5 × 1.4 × 1400 mm. The CFRP sheet was modelled as a deformable shell part, following the contour of the LVL beam. The width of the sheet measured 150 mm for the B and C series and 222.5 mm for the D series. The steel guide plate was conceptualized as a “Discrete Rigid” [16] body in three-dimensional space by extruding a shell of dimensions 10 × 100 mm. The extrusion value for the visualization was 22.5 mm. Cross-sections for every configuration of strengthening as well as a side view of selected model are depicted in Figure 3.

The mesh generation process was executed on suitably partitioned parts, distinguishing between rectilinear constituents and those of curvilinear geometry. The assumed approximate mesh size for all components was 10 mm. Its densification was applied in curved zones and the vicinity of cuttings, locally augmenting the number of elements on chosen contours. The assumed element type for solid elements was C3D8R (an eight-node linear brick with reduced integration and hourglass control); for shell elements, S4R (a four-node doubly curved thin or thick shell with reduced integration and hourglass control), and for discrete rigid elements, R3D4 (a four-node 3D bilinear rigid quadrilateral) [16]. A hexagonal configuration of the finite element shape, derived from the medial axis algorithm, was applied for elements possessing curvatures. 

The interaction between steel guide plates and the surfaces of the LVL beam was accommodated using the “surface-to-surface contact (Standard)” methodology. The contact characteristics account for the incapability of parts penetration via the “Hard” contact option in the normal direction, while in the tangential direction, a friction coefficient of 0.3 was incorporated, utilizing the “penalty” sub-option. A “Tie” constrain method established connection between components such as veneer–glue, veneer–sheet, and glue–laminate. The CFRP laminate inside the groove was modelled using the “Embedded region” constraint.

**Figure 4:**
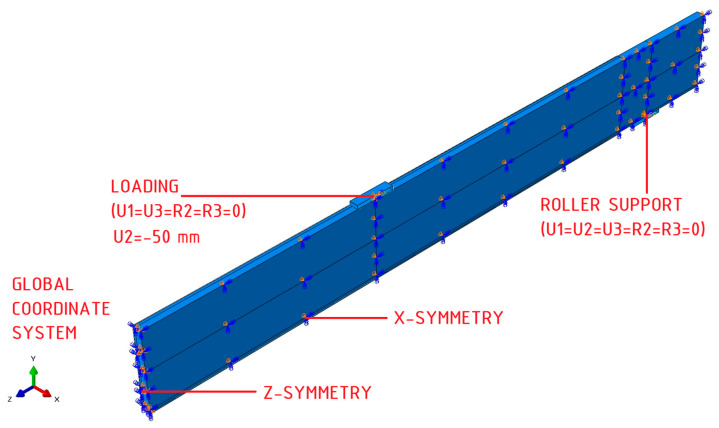
Boundary conditions.

The boundary conditions adopted are presented in Figure 4. The system’s symmetry in the direction of the *x* and *z* axes, the global coordinate system, is considered. Moreover, at the reference points on the support and load guide plate, blockage of all degrees of freedom was assumed except for the rotation about the *x* axis (U1 = U2 = U3 = R2 = R3 = 0, R1 ≠ 0), which was allocated in the initial step. This condition matches with the ability to rotate of the steel plate during experimental tests. The load on the beam was implemented by the displacement of the steel guide plate located on the upper surface of laminated veneer lumber beam; a displacement of −50 mm relative to the *y* axis was applied in the Loading step.

The computations were executed within the static domain. To achieve this, a step categorized as “Static, General” was created. The initial, minimal, maximal, and maximum number of increments were adapted to ensure the solution’s accuracy. The deflection values were read at the nodal point located at the mid-span of the beam. The value of the load was read from a reference point on the steel plate.

### 3.2. Material Models

Behavioral models of materials were developed based on findings from previous experimental research [32], supplemented with manufacturer data [36,37,38,39] and studies conducted by other investigators on identical materials [19]. The constant elements in the examination were the material models of CFRP composites and adhesives. The variable parameter under consideration was the material model for laminated veneer lumber.

A linearly elastic–perfectly plastic material model was implemented for adhesives and CFRP sheets. An orthotropic material model was utilized for carbon fiber reinforced polymer laminates. In the case of laminated veneer lumber, two models were examined: a linearly elastic one and a linearly elastic–perfectly plastic one featuring Hill’s functions.

Selected mechanical properties of LVL, composites, and resins are presented in Table 1, Table 2, Table 3 and Table 4.

The values presented in Table 4 for composite materials based on [36,37] have been evaluated under tensile tests and should be always utilized within these circumstances. Hence, the mechanical characteristics of bonded composite materials on external faces within the compression zone have been altered. The compressive strength of the reinforcement in the compressive zone was assumed to be equal to the strength of the adhesive used to its application, predicated on the supposition that, following its failure, the composite would cease to partake in the load transmission. The modulus of elasticity was estimated as the arithmetic mean of the adhesive and the composite sheet. This premise aligns with the findings from the empirical investigation. This also represents a safe approach, incorporating the sheet’s flexible characteristics. Initial analyses exposed that presuming homogeneous properties in the compressive and tensile zones for the composite would lead to a significant overestimation of the outcomes.

The orthotropic models in Abaqus were determined using the elastic characteristics utilizing the “Engineering constants” sub-function. The nine variables delineated in Table 2 and Table 3 are incorporated therein. The Hill function was calculated using Equation (1) and incorporated in the “Potential” option:

(1) $$f\left(\sigma \right)=\sqrt{F{\left(\sigma_{22}-\sigma_{33}\right)}^{2}+G{\left(\sigma_{33}-\sigma_{11}\right)}^{2}+H{\left(\sigma_{11}-\sigma_{22}\right)}^{2}+2N\tau_{12}^{2}+2M\tau_{13}^{2}+2L\tau_{23}^{2}}$$

where *σ_ij_* and *τ_ij_* refer to the stress tensors *σ*, and the six constants can be estimated via Equations (2) and (3):

(2) $$F=\frac{1}{2}\left(\frac{1}{R_{22}^{2}}+\frac{1}{R_{33}^{2}}-\frac{1}{R_{11}^{2}}\right),\;G=\frac{1}{2}\left(\frac{1}{R_{33}^{2}}+\frac{1}{R_{11}^{2}}-\frac{1}{R_{22}^{2}}\right),H=\frac{1}{2}\left(\frac{1}{R_{11}^{2}}+\frac{1}{R_{22}^{2}}-\frac{1}{R_{33}^{2}}\right),$$

(3) $$N=\frac{3}{{2R}_{12}^{2}},M=\frac{3}{{2R}_{13}^{2}},L=\frac{3}{{2R}_{23}^{2}}.$$

These constants were estimated utilizing equations from the Abaqus program textbook [16]. Presented below are the outcomes of estimations, derived from the data in Table 1, for laminated veneer lumber:

(4) $$R_{11}=\frac{{\bar{\sigma }}_{11}}{\sigma^{0}}=\frac{58.5\;MPa}{58.5\;MPa}=1.0,$$

(5) $$R_{22}=R_{33}=\frac{{\bar{\sigma }}_{22}}{\sigma^{0}}=\frac{{\bar{\sigma }}_{33}}{\sigma^{0}}=\frac{9.5\;MPa}{58.5\;MPa}=0.162,$$

(6) $$R_{12}=R_{13}=\frac{{\bar{\sigma }}_{12}}{\tau^{0}}=\frac{{\bar{\sigma }}_{13}}{\tau^{0}}=\frac{4.6\;MPa}{33.77\;MPa}=0.136,$$

(7) $$R_{23}=\frac{{\bar{\sigma }}_{23}}{\tau^{0}}=\frac{2.6\;MPa}{33.77\;MPa}=0.077,$$

where *τ*^0^ = *σ*^0^/√3. Estimated constants are summarized in Table 5.

In subsequent simulations, numerical models were designated according to the “Series Name–Finite Element Method–Material model” convention; for instance, A-FEM-E signifies a numerical model of Series A adopting a linearly elastic behavior model for laminated veneer lumber; A-FEM-E-P denotes a numerical model of Series A implementing a linearly elastic–perfectly plastic LVL material model with Hill’s yield criterion.

## 4. Results and Discussion

The numerical simulation’s outcomes are depicted in relation to load-bearing capacity, stiffness, deflection, and stresses. Maps are represented for half of the entire beam. To accomplish this, the findings obtained from 1/4 of the beam were mirrored based on a locally established coordinate system.

### 4.1. Load-Bearing Capacity

**Figure 5:**
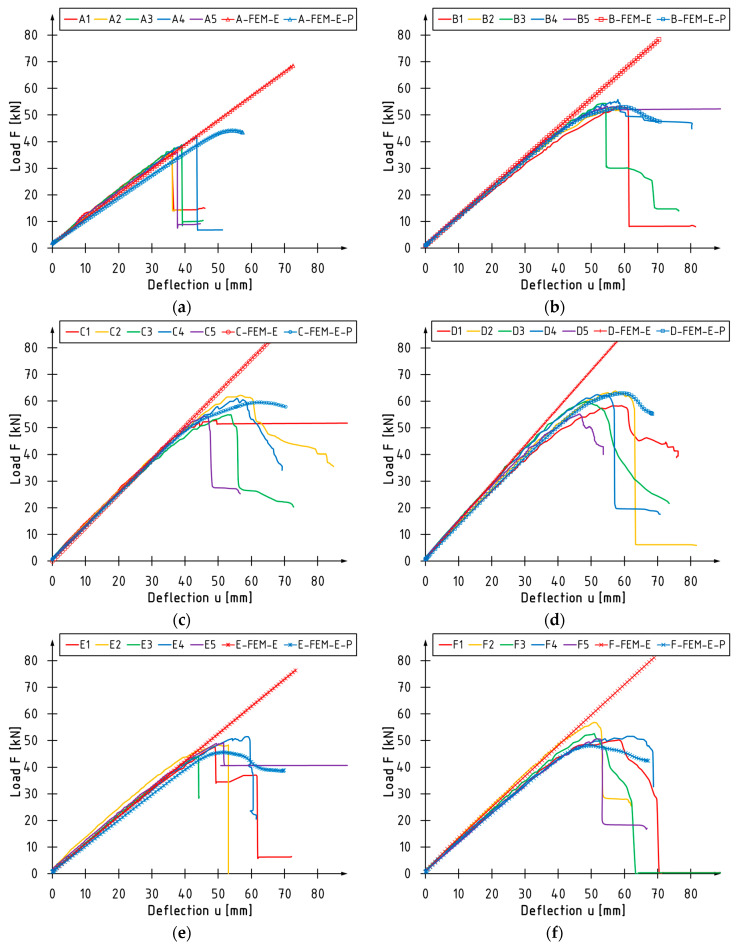
Load versus deflection curves for series: (**a**) A; (**b**) B; (**c**) C; (**d**) D; (**e**) E; (**f**) F.

In Figure 5, the load versus deflection curves for the examined experimental series are depicted, also including curves derived from numerical models. The results for the linearly elastic material are plotted in red with markers, while those for the elastic–plastic material model for LVL are marked in blue with indicators. 

The behavior of unstrengthened beams is accurately described by a linear material model, a methodology the authors strongly suggest for such components. Within the context of the elastic–plastic model, the beams’ stiffness is considered inferior compared to the experimental investigations. In the final phase of the bending test simulations, the plasticization of LVL occurred in the compressive zone. This phenomenon was not documented in the experimental research.

In contrast, for strengthened beams, the elastic–perfectly plastic material model rather than linearly elastic material model should be utilized for the laminated veneer lumber. This is naturally linked to the change in failure mode from brittle fracture to more ductile modes. For beams strengthened with sheets, it correctly predicts the appearance of a plastic zone, the occurrence of a peak at the maximum loading force and a decrease in force once this is exceeded. Similar conclusions can be deduced from the analysis of charts for beams strengthened with CFRP laminates. However, it is important to note that the peak of the maximum force is lower than the curves observed in the experiments. This is likely due to the inaccurate modelling of the properties of resin adhesives, for which data from the manufacturer’s catalogues were used. These characteristics were probably superior, and given the extensive volume of the adhesive joint, in comparison to series B, C and D, this should result in enhanced load-bearing capacity of the cross-section.

Fluctuations in the course of the curves, visible in the final part of bending tests, depend on the failure mode. Significant drops in loading force were mainly caused by failure in the tension zone due to cracking of LVL, or the debonding or rupture of CFRP, while the ductile modes correspond to crushing of LVL in the compressive zone.

Table 6 compares the experimental, theoretical, and numerical research outcomes. The experimental values were obtained from [32]. Theoretical values were derived from [31], estimated using the transformed cross-section method. Numerical results are displayed for the elastic–plastic model of laminated veneer lumber. The comparison was carried out using the maximum values of loading force and their corresponding deflection values at the midspan. In the case of maximum loading force, the most significant difference was observed between unstrengthened beams and those strengthened with carbon laminates. For beams strengthened with sheets, these differences do not exceed 4%. Conversely, a more significant deviation was obtained in the instance of deflection values. They are usually larger than those from experimental studies. The most significant differences were noted for the A and C series beams, 37% and 18%, respectively. In terms of theoretical values, they are marginally more accurate to reality for series A, E, and F than the numerical ones.

### 4.2. Stiffness

**Figure 6:**
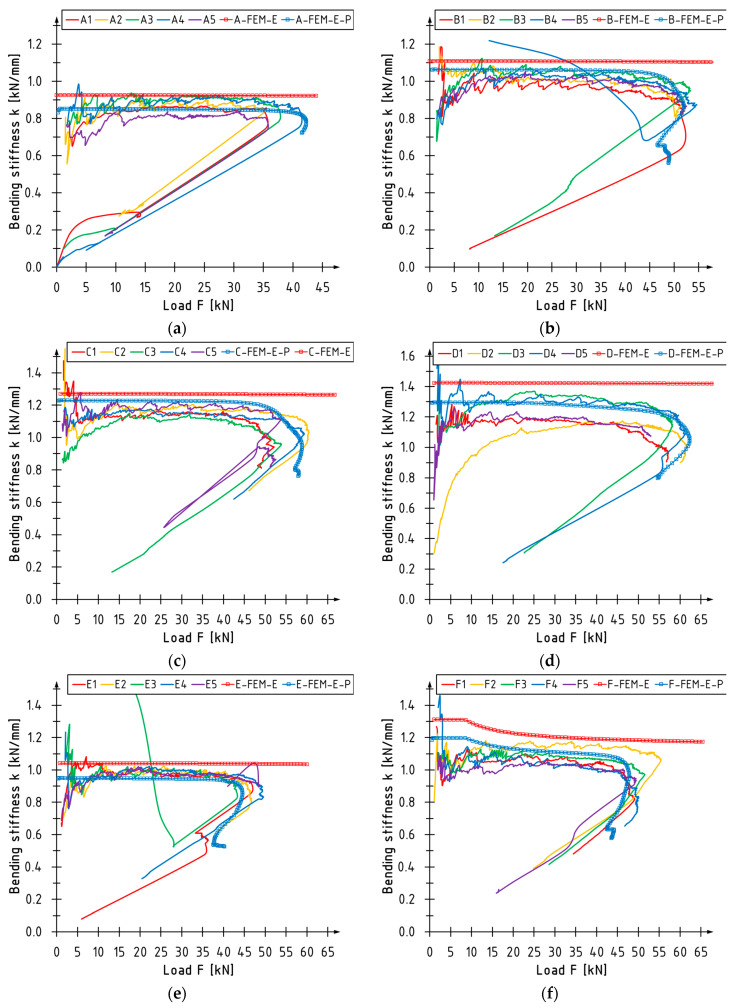
Stiffness changes versus loading for series: (**a**) A; (**b**) B; (**c**) C; (**d**) D; (**e**) E; (**f**) F.

Figure 6 shows graphs indicating the change in bending stiffness coefficient *k* in function of the loading force F. The bending stiffness was calculated as the ratio between the loading force and the midspan deflection values, which aligns with [31]. The results for the linearly elastic material model are plotted in red with markers, while those for the elastic–perfectly plastic material model for LVL are marked in blue with indicators. The linear model outputs slightly exaggerated results in each of the inspected strengthening scenarios. The curves that match the elastic–plastic model accurately reflect the behavior of real-world elements. The decrease in stiffness and the distortion of the curves is visible here. The decrease in stiffness during bending is caused by damages in LVL and the strengthening system.

Table 7 presents a comparison of numerical and experimental values of bending stiffness. In the case of the linear model, discrepancies vary between 3 and 16%. The slightest deviation was noted for unstrengthened beams and those strengthened with laminates bonded into grooves. In the case of the elastic–plastic model, these values fall within the range of −7 to 7%. In this instance, the most significant discrepancies were observed for beams strengthened with carbon laminates, representing both the range’s lower and upper limits. According to the authors, these differences can be minimized by modifying the parameters of the adhesive material. However, it is worth noting that this model accurately predicts the bending stiffness of beams reinforced with CFRP sheets, the maximum discrepancy being 4% for the C series. Comparable precision was achieved for the stiffness estimates of smaller beams strengthened with sheets [40].

### 4.3. Stress

In all the maps featured in this subsection, the stresses have been expressed in megapascals.

**Figure 7:**
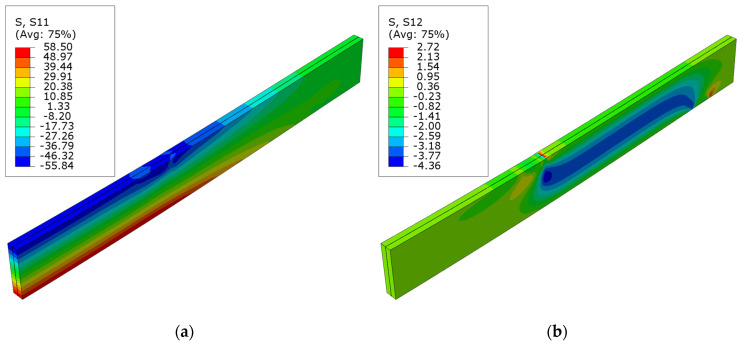
Stress distribution in LVL at ultimate load, for series A: (**a**) normal S11 stress; (**b**) shear S12 stress.

Figure 7 presents the normal stress S11 and shear stress S12 for the unstrengthened beam. Minor fluctuations in the stress distribution can be observed at the point where the concentrated force was applied and on the support; for instance, local alterations in compressive stress on the upper surface (S11). The maximum normal stress equals the assumed plasticity point of 58.5 MPa and occurs in the highly tensioned fibers.

**Figure 8:**
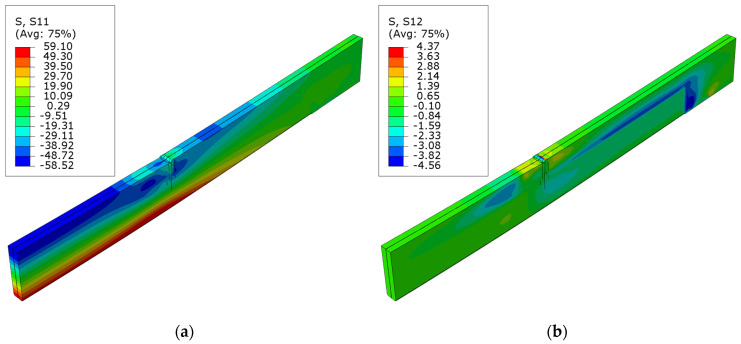
Stress distribution in LVL at ultimate load for series B: (**a**) normal S11 stress; (**b**) shear S12 stress.

**Figure 9:**
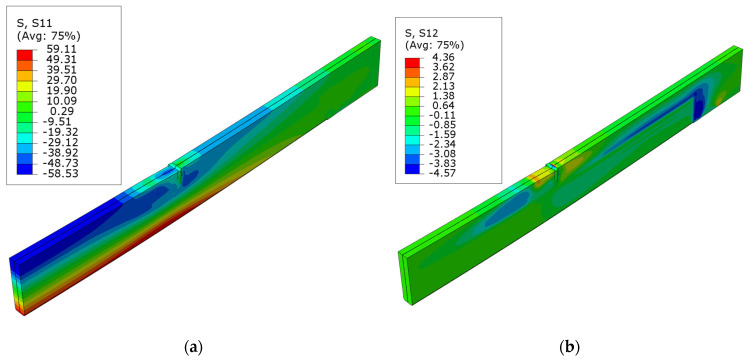
Stress distribution in LVL at ultimate load for series C: (**a**) normal S11 stress; (**b**) shear S12 stress.

**Figure 10:**
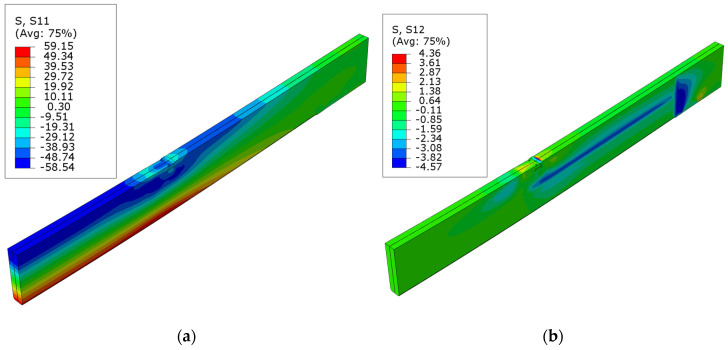
Stress distribution in LVL at ultimate load for series D: (**a**) normal S11 stress; (**b**) shear S12 stress.

The diagrams of normal S11 and shear S12 stress for beams strengthened with carbon fiber sheets can be seen in Figure 8, Figure 9 and Figure 10. In this instance, there was a significant decrease in the shear forces at the site where the sheet was applied. The positioning of the composite well depicts the layout of these stresses. This occurrence is also observed in the D series, where a sheet in the compression zone was designed with modified mechanical strengths. In the case of normal stresses, as the level of cross-sectional reinforcement ratio grows, there is an increase in the area of maximum tensile and compressive stresses. The disturbances in the maps can be seen here at the point where the load is applied to the surface of the upper beam. This is related to damage from veneer crushing. The maximum tensile stress is roughly 59.1 MPa. This value exceeds the material model’s assumed yield strength of the LVL. As other researchers have documented, it can be inferred that there is a higher increase in tensile stresses compared to an unreinforced element. This arises from the LVL bound by the composites bonded to the external surfaces.

**Figure 11:**
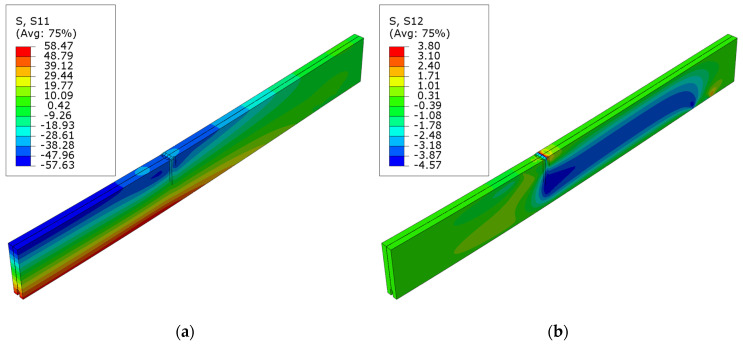
Stress distribution in LVL at ultimate load for series E: (**a**) normal S11 stress; (**b**) shear S12 stress.

Figure 11 shows maps of the normal S11 and shear S12 stresses in beams strengthened with laminates inserted into grooves. Here, it can be observed that the tensile stresses slightly exceed the compressive stresses upon reaching the maximum load force. This could suggest that the cross-section is not adequately reinforced to utilize the veneer’s compressive strength reserves. This is also associated with local veneer damage due to grooves being hollowed out from the bottom and the positioning of the reinforcement. The values of S11 stress are also slightly lower than the assumed yield strength.

**Figure 12:**
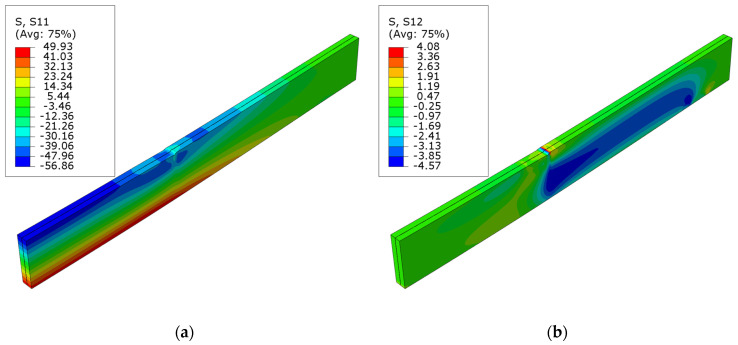
Stress distribution in LVL at ultimate load for series F: (**a**) normal S11 stress; (**b**) shear S12 stress.

Figure 12 presents the normal S11 and shear S12 stress maps for beams strengthened with laminates bonded to their lower surface. It is worth noting that the maximum tensile stresses in the LVL are lower than the yield strength assumed in the model. Unlike bonded laminates in grooves, more significant stresses are present in the compressed area of the veneer. In future simulations, the possibility of combining the CFRP laminate with bonded sheets in critical areas—the anchoring zone and the load axis—should be considered.

**Figure 13:**
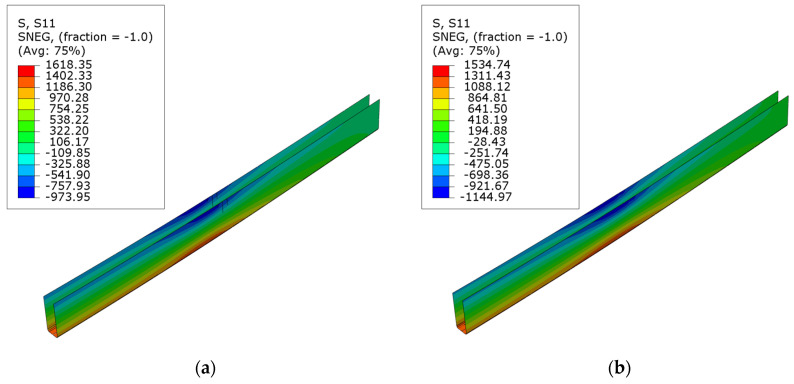

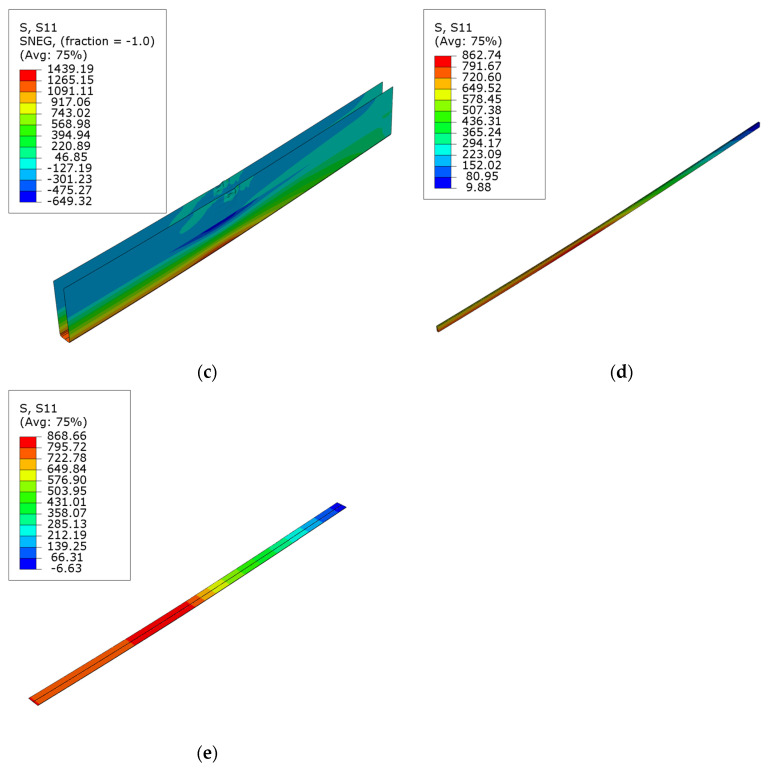
Normal S11 stress distribution in CFRP composites on ultimate load, for series: (**a**) B; (**b**) C; (**c**) D; (**d**) E; (**e**) F.

The normal stress maps for composite reinforcement are depicted in Figure 13. The maximum tensile stresses for carbon sheets were 1618.35, 1534.74, and 1439.19 MPa for series B, C, and D beams, respectively. The stresses reduced as the ratio of cross-sectional reinforcement increased. However, this dependency is not linear. Comparable maximum tensile stress values were recorded for carbon laminates; specifically, 862.74 and 868.66 MPa for the E and F series, respectively. The composite exhibits similar tensile stress if the laminate is applied internally or externally to the cross-section. Therefore, placing the reinforcement on the external surfaces is more beneficial, as it is a more effective solution. However, it is worth noting that the utilization of the composite’s tensile strength is minimal. Depending on its type, it amounts to a maximum of 32% for sheets and 40% for laminate of the values provided by the manufacturer [36,37]. These values align with those obtained from measurements using an ARAMIS-type optical system [30]. Similar results were also achieved by using the transformed cross-section method [31]. 

It must be highlighted here that, despite the stresses raised during testing being much lower than the manufacturer declared, the composite material—specifically the CFRP sheet—was damaged. However, this damage was associated with the failure of the laminated veneer lumber and its subsequent effects. For instance, the buckling of the veneer in the compression zone coincided with the buckling or shearing of the sheet, and the cracking of the veneer in the tensile zone was accompanied by local sheet rupture, when sharp veneer pieces pierced it. However, it is worth noting that such a phenomenon only occurred in the case of a single sheet layer. Therefore, it could be inferred that two layers are enough to bond the veneer and allow its gradual degradation via plasticization. 

Additionally, the highest stress in the composite is reached at the location of the concentrated force application, irrespective of the analyzed strengthening layout. This implies that solely concentrating on analyzing deformations and stresses in the section in the middle of the beam span is not the correct method. The analysis should also include the near loading zones.

Upon analysis of the composite sheets’ stress maps, it can be observed that this material does not engage in load transfer in the support zone on the side surfaces (B and C series). It is worth verifying the feasibility of removing it from this section of the beam to achieve a more cost-effective solution. Similar conclusions can be drawn by analyzing the compressive stresses in the sheet (series D). In this instance, removing the sheet from the compression zone and substituting it with steel components is recommended.

### 4.4. Failure Mode and Deformations

**Figure 14:**
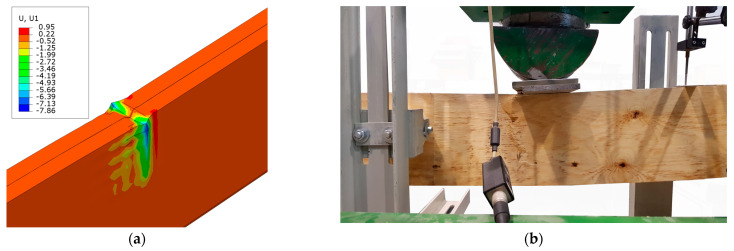
(**a**) Typical deformation of LVL at the concentrated load; (**b**) corresponding failure mode.

Figure 14 shows a typical LVL deformation (displacement along the *x* axis) derived from the numerical model at the point of application concentrated force. Next to it, the corresponding failure mode is presented. The values on the diagram are specified in millimeters. This deformation is consistent and typical of the LVL damage observed during experimental studies for all strengthening configurations. The destruction of compressed veneer typically results from the buckling of its outer layers. This has been presented in the works of [27,28,29,30,31]. Similar local damage, but on a much smaller scale, was also recorded on the supports.

**Figure 15:**
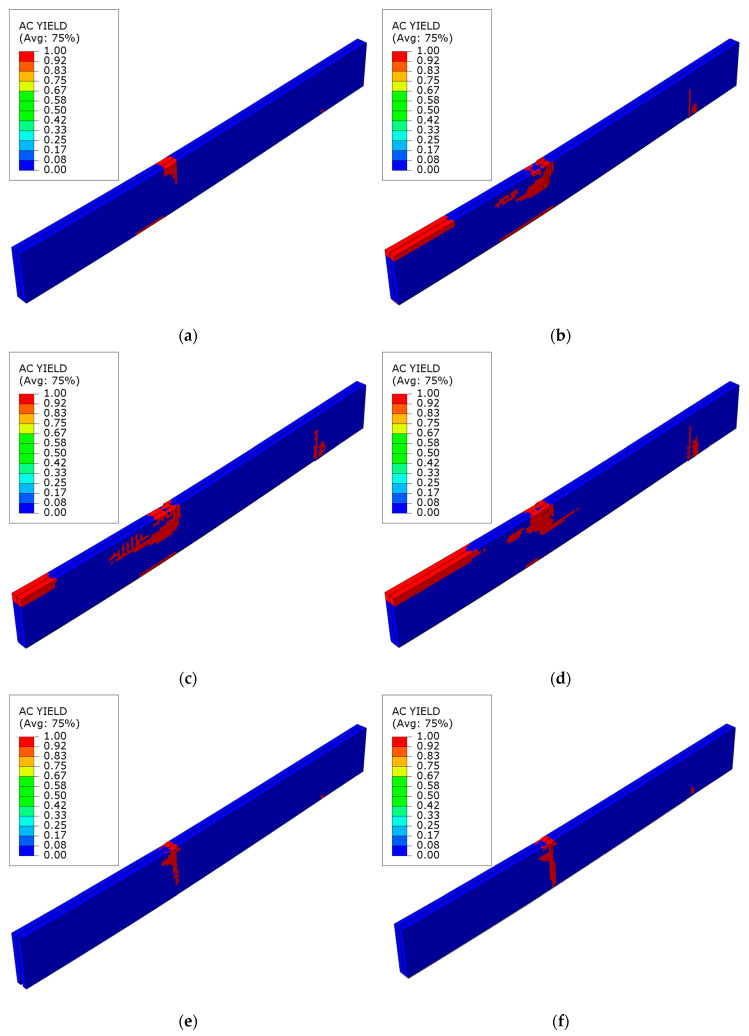
Plasticity mode of LVL at ultimate load for series: (**a**) A; (**b**) B; (**c**) C; (**d**) D; (**e**) E; (**f**) F.

Figure 15 illustrates the plasticization mode of the laminated veneer lumber at the point of maximum loading force. For an unstrengthened beam, damage initially happens at the place of application of the concentrated force, within the areas of compression and tension. A small area can also be seen at the support location. This is an idealized phenomenon, and it does not consider the potential occurrence of material flaws. The strengthened beams exhibit similar behavior in the initial phase. However, these areas expand with increasing load due to the presence of reinforcement. In the case of beams strengthened with sheets, there is a noticeable participation of the compressed area in the middle of the span and at the point where the sheet ends on the support. In the case of laminates, only the area within the concentrated force across its entire height was subjected to plasticization. Detailed description of failure modes for all analyzed reinforcement cases can be found in the referenced works [27,32].

**Figure 16:**
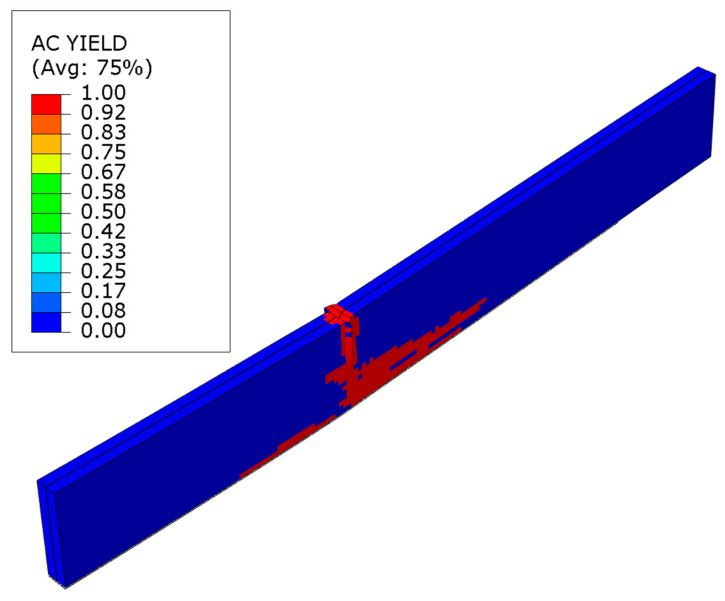
Plasticity mode of LVL post ultimate load for series F.

Notably, a plastic hinge typically forms when the maximum load force is surpassed. This stems from the propagation of damage at the site where concentrated force is applied, the crushing of the veneer. Substantial alterations subsequently transpire in the methodology of the veneer’s plasticization. The deformations in the central sections are diminishing. They are increasing along the loading axis. Figure 16 demonstrates this for a beam strengthened with laminate bonded to the lower surface. A significant plasticity within the tensile zone is evident, as described in the work [29].

These results suggest that to enhance the efficiency of the presented reinforcement configurations, the primary emphasis should be placed on the compression and tension zones within the axis of applied concentrated load.

## 5. Conclusions

This paper presents a numerical analysis of beams strengthened with carbon fiber-reinforced sheets and laminates. The results of the numerical analysis were compared with experimental and theoretical studies. 

It was determined that a linearly elastic material model for laminated veneer lumber should be applied to describe the behavior of unstrengthened beams. This is related to the absence of plasticization in the compression and tension zones; the beam’s failure results from brittle cracking. For strengthened beams, the elastic–plastic model should be used for LVL. This is because, in both the compression and tension zones, the reinforcement causes the LVL to plasticize. 

In numerical modelling, the need to reduce the mechanical properties of composite sheets bonded to external surfaces in the compression zone was confirmed. The appropriate solution for the examples shown was to reduce the yield strength to the lesser of the adhesive or composite sheet strength. Stiffness should be taken to mean averaged. In the case of composites that are only subjected to tensile stress, adopting elastic material models appears to be sufficient. This is a direct result of the stress during the test being much lower than the limit stress.

The adhesive properties can be ignored when modelling tensile zone reinforcement with flexible, elastic fabrics applied with a thin adhesive bond. No worsening in results was caused by this assumption. In other cases, the E- and F-series beams, adhesive properties significantly impacted on the performance of the flexural member.

Analysis of the stresses and plasticity in the LVL indicates that to achieve higher reinforcement efficiencies; the primary focus should be on additional reinforcing the compression zone. The use of steel elements and mechanical fasteners is a suggestion here.

Future research is planned to test the applicability of the Hashin damage model to the description of the failure of composite materials (carbon sheets and laminates). There is a need for conducting experimental research in this area, which has not been conducted by the authors. Material tests should also be carried out for epoxy-based adhesives. Finally, modelling the veneer–veneer and veneer–composite joint with an epoxy-based adhesive requires more attention.

## Figures and Tables

**Table 1:** Selected mechanical properties of laminated veneer lumber [32,35].

Parameter	Value
Compressive strength parallel to the grain *f_c_*_,0_ [MPa]	58.5
Compressive strength perpendicular to the grain *f_c_*_,90,*edge*_ [MPa]	9.5
Bending strength (edgewise condition) *f_m_*_,0,*edge*_ [MPa]	56.7
Shear strength parallel to the grain in edgewise condition *f_v,_*_0,*edge*_ [MPa]	4.6
Shear strength parallel to the grain in flatwise condition *f_v_*_,0,*flat*_ [MPa]	2.6

**Table 2:** Orthotropic properties of laminated veneer lumber [19,32,35].

Modulus of Elasticity [MPa]			Shear Modulus [MPa]			Poisson’s Ratio [-]		
E1	E2	E3	G12	G13	G23	ν12	ν13	ν23
13,000	430	430	600	600	96	0.48	0.48	0.48

**Table 3:** Orthotropic properties of carbon fiber reinforced polymer laminates [32,37].

Modulus of Elasticity [MPa]			Shear Modulus [MPa]			Poisson’s Ratio [-]		
E1	E2	E3	G12	G13	G23	ν12	ν13	ν23
200,000	7100	7100	2730	2730	2730	0.3	0.3	0.3

**Table 4:** Selected mechanical and physical properties of adhesives and composites [36,37,38,39].

Parameter	S&P C-Sheet 240	S&P C-Laminate	S&P Resin 55 HP	S&P Resin 220	S&P C-Sheet 240 + S&P Resin 220 (Compression)
Modulus of elasticity [N/mm^2^]	265,000	200,000	3200	7100	134,100
Tensile strength [N/mm^2^]	5100	2200	-	-	5100
Compressive strength [N/mm^2^]	-	-	100	70	100
Bending strength [N/mm^2^]	-	-	85.33	41.91	-
Elongation at rupture [%]	1.7	1.6	1.73	-	-
Thickness [mm]	0.333	-	-	-	0.333

**Table 5:** Constants assumed for analysis.

*R*11	*R*22	*R*33	*R*12	*R*13	*R*23
1.0	0.162	0.162	0.136	0.136	0.077

**Table 6:** Comparison of experimental [32], theoretical [31], and numerical values of maximum load and corresponding midspan deflection.

Series	Maximum Load [kN]				Deflection Corresponding to Maximum Load [mm]		
	Experimental	Theoretical	Numerical	Numerical/ Experimental	Experimental	Numerical	Numerical/ Experimental
A	37.91	39.04	42.25	1.11	39.57	54.22	1.37
B	53.96	48.00	51.98	0.96	56.24	59.61	1.06
C	57.13	56.44	58.80	1.03	52.89	62.37	1.18
D	59.97	62.97	62.32	1.04	52.59	59.28	1.13
E	48.32	45.73	44.87	0.93	51.27	51.19	1.00
F	52.41	50.51	47.80	0.91	54.57	49.81	0.91

**Table 7:** Comparison of experimental [32] and numerical values of stiffness coefficient *k* obtain for elastic and elastic–perfectly plastic material models for LVL.

Series	Elastic Model of LVL [kN/mm]			Elastic–Perfectly Plastic Model of LVL [kN/mm]		
	Experimental	Numerical	Numerical/ Experimental	Experimental	Numerical	Numerical/ Experimental
A	0.900	0.926	1.03	0.900	0.851	0.95
B	1.036	1.109	1.07	1.036	1.062	1.03
C	1.182	1.270	1.07	1.182	1.228	1.04
D	1.283	1.424	1.11	1.283	1.295	1.01
E	1.026	1.042	1.02	1.026	0.949	0.93
F	1.092	1.263	1.16	1.092	1.173	1.07

## Data Availability

All data are available in the article.
